# Supplementary material for: Public knowledge, attitudes and practices towards COVID-19: A cross-sectional study in Malaysia
Source: PLoS One. 2020 May 21;15(5):e0233668. doi: 10.1371/journal.pone.0233668 (PMC7241824; doi:10.1371/journal.pone.0233668)
Supplement: Attachment — Submitted filename: Response to Reviewers.docx [file pone.0233668.s002.docx]

Dear Editor,

Thank you for conveying the reviewer’s comments on our article entitled “Public knowledge, attitudes and practices towards COVID-19: A cross-sectional study in Malaysia” (PONE-D-20-11518). We have reviewed the comments and provided more information to address their concerns. This letter will provide you with a point-by-point response as well as details on where the corrections/ additions were made on the manuscript. We have also highlighted the changes in a copy of the manuscript, as requested.

Reviewer report:

Reviewer 1.

1. **The sampling for this study was how to elect? Will there be deviations in different social media?**

The sampling for this study was based on a convenience sample of professional and personal networks of the researchers. Facebook and Whatsapp were chosen as the two most widely-used communication platforms in Malaysia. The choice to disseminate the survey among different social media applications was to reach participants of different age groups (Pages 5 & 6, lines 126-129).

2. **How to explain the difference in results between people over 50 and people of other ages.**

The difference in results for people over 50 years of age is addressed in terms of risk perception (Page 15, lines 314-315) and cultural norms (Page 17, lines 356-359) affecting practice in the COVID-19 pandemic.

3. **How does income and occupation affect COVID-19.**

Income and occupation play a role in knowledge levels as well as attitudes in the COVID-19 pandemic whereby those with lower income may have limited access to timely information on the virus (Page 15, lines 315-317) and those working in the public sector showed higher percentages of positive attitudes toward efforts made in battling COVID-19 (Page 17, lines 344-348). Income also affects practice in that those with higher incomes in terms of compliance to health recommendations and fear and control in pandemic situations (Page 17, lines 359-361).

4. **The following references should be discussed in the revision text.**

**Cao JL, Hu XR, Tu WJ., & Liu Q. (2020). Clinical Features and Short-term Outcomes of 18 Patients with Corona Virus Disease 2019 in Intensive Care Unit. Intensive Care Medicine, DOI: 10.1007/s00134-020- 05987-7.**

**Cao JL, Tu WJ, Hu XR, & Liu Q. (2020). Clinical Features and Short-term Outcomes of 102 Patients with Corona Virus Disease 2019 in Wuhan, China. Clinical Infectious Diseases, DOI: 10.1093/cid/ciaa243/ 5814897.**

The references have been added to the manuscript (Page 3, line 74-75 and page 15, line 314-315) to explain the transmission of the disease and the higher risk levels for older patients.

5. **What are the reasons for the lack of masks? Any good suggestions?**

The low use of face masks among the Malaysian public was due to societal norms – it is not common for Malaysians to use face masks when they feel sick (Page 17, lines 366-369).

It is also mentioned that the Ministry of Health Malaysia did not encourage the use of medical face masks among the public in order to ensure sufficient supply to healthcare workers (Page 18, lines 372-375).

On the other hand, the lack of face masks available for sale in the country were due to limited global supply (Page 18, lines 370-372).
